# Supplementary material for: FGF7 promotes load-bearing tendon regeneration and suppresses fibrosis
Source: Nat Commun. 2025 Dec 27;17:708. doi: 10.1038/s41467-025-67355-7 (PMC12820144; doi:10.1038/s41467-025-67355-7)
Supplement: Supplementary file 1 — Reporting summary [file 41467_2025_67355_MOESM1_ESM.pdf]

nature portfolio

Corresponding author(s): Xiao Chen,Zi Yin

Last updated by author(s): Nov 21, 2025



# Reporting Summary

Nature Portfolio wishes to improve the reproducibility of the work that we publish. This form provides structure for consistency and transparency in reporting. For further information on Nature Portfolio policies, see our Editorial Policies and the Editorial Policy Checklist.

## Statistics

For all statistical analyses, confirm that the following items are present in the figure legend, table legend, main text, or Methods section.

| n/a | Confirmed | |
|---|---|---|
| ☐ | ☒ | The exact sample size (*n*) for each experimental group/condition, given as a discrete number and unit of measurement |
| ☐ | ☒ | A statement on whether measurements were taken from distinct samples or whether the same sample was measured repeatedly |
| ☐ | ☒ | The statistical test(s) used AND whether they are one- or two-sided<br>*Only common tests should be described solely by name; describe more complex techniques in the Methods section.* |
| ☒ | ☐ | A description of all covariates tested |
| ☒ | ☐ | A description of any assumptions or corrections, such as tests of normality and adjustment for multiple comparisons |
| ☐ | ☒ | A full description of the statistical parameters including central tendency (e.g. means) or other basic estimates (e.g. regression coefficient) AND variation (e.g. standard deviation) or associated estimates of uncertainty (e.g. confidence intervals) |
| ☐ | ☒ | For null hypothesis testing, the test statistic (e.g. *F*, *t*, *r*) with confidence intervals, effect sizes, degrees of freedom and *P* value noted<br>*Give P values as exact values whenever suitable.* |
| ☒ | ☐ | For Bayesian analysis, information on the choice of priors and Markov chain Monte Carlo settings |
| ☒ | ☐ | For hierarchical and complex designs, identification of the appropriate level for tests and full reporting of outcomes |
| ☒ | ☐ | Estimates of effect sizes (e.g. Cohen's *d*, Pearson's *r*), indicating how they were calculated |

*Our web collection on statistics for biologists contains articles on many of the points above.*

## Software and code

Policy information about availability of computer code

| | |
|---|---|
| Data collection | No custom code or software was used to collect the existing sequence data |
| Data analysis | Data analyses were performed using R system software (version 4.0.5; https://www.r-project.org/), along with packages from the Bioconductor project or original R code. This paper does not report custom code. |

For manuscripts utilizing custom algorithms or software that are central to the research but not yet described in published literature, software must be made available to editors and reviewers. We strongly encourage code deposition in a community repository (e.g. GitHub). See the Nature Portfolio guidelines for submitting code & software for further information.

## Data

Policy information about availability of data

All manuscripts must include a data availability statement. This statement should provide the following information, where applicable:

- Accession codes, unique identifiers, or web links for publicly available datasets
- A description of any restrictions on data availability
- For clinical datasets or third party data, please ensure that the statement adheres to our policy

The raw sequence data of Single-cell RNA-sequencing and RNA-Seq reported in this paper have been deposited in the Genome Sequence Archive 43 in National Genomics Data Center 44, China National Center for Bioinformation/Beijing Institute of Genomics, Chinese Academy of Sciences (GSA:CRA019874; GSA:CRA019760) that are publicly accessible at https://ngdc.cncb.ac.cn/gsa/browse/CRA019874 and https://ngdc.cncb.ac.cn/gsa/browse/CRA019760. Additional datasets used in this

study are available: Metascape (http://metascape.org/), GeneOntology (http://geneontology.org/), KEGG(htpps://www.kegg.jp/) and GSEA(https://www.gseamsigdb.org/gesa/index.jsp). Source data are provided with this paper. Source data are provided with this paper.

## Research involving human participants, their data, or biological material

Policy information about studies with human participants or human data. See also policy information about sex, gender (identity/presentation), and sexual orientation and race, ethnicity and racism.

| | |
|---|---|
| Reporting on sex and gender | Sex was determined based on self-reporting. Sex-related data were obtained from the patients' clinical records with their consent. This study recorded the sex information of 75 individuals. Due to the occurrence of tendon fibrosis across different physiological sexes, our study did not include a comparative analysis based on physiological sex. The study participants had a mean age of over 50, with a female majority. |
| Reporting on race, ethnicity, or other socially relevant groupings | No socially constructed or socially relevant categorization variable(s) were used in my manuscript. |
| Population characteristics | We collected information on patients' age, medical history, and other relevant details, primarily from patients with rotator cuff disorders or undergoing ACL reconstruction from China. |
| Recruitment | Patients were recruited from the Second Affiliated Hospital of Zhejiang University School of Medicine,include individuals with rotator cuff tendon disease. Healthy hamstring tendon samples were obtained from patients undergoing ACL reconstruction. |
| Ethics oversight | Ethics Committee of the second Affiliated Hospital, School of Medicine, Zhejiang University approved the study protocol. |

Note that full information on the approval of the study protocol must also be provided in the manuscript.

# Field-specific reporting

Please select the one below that is the best fit for your research. If you are not sure, read the appropriate sections before making your selection.

☒ Life sciences ☐ Behavioural & social sciences ☐ Ecological, evolutionary & environmental sciences

For a reference copy of the document with all sections, see nature.com/documents/nr-reporting-summary-flat.pdf

# Life sciences study design

All studies must disclose on these points even when the disclosure is negative.

| | |
|---|---|
| Sample size | No statistical method was employed to pre-determine the sample size and the sample size was established based on previous experiments with a similar design. |
| Data exclusions | No data were excluded from the analyses |
| Replication | The experimental findings were reproducible and were replicated at least for three times |
| Randomization | Each sample is assigned a unique number prior to grouping and is subsequently organized through a random grouping method. |
| Blinding | The experiment operator is distinct from the experiment designer, and the operator is unaware of the grouping arrangement. |

# Reporting for specific materials, systems and methods

We require information from authors about some types of materials, experimental systems and methods used in many studies. Here, indicate whether each material, system or method listed is relevant to your study. If you are not sure if a list item applies to your research, read the appropriate section before selecting a response.

### Materials & experimental systems

| n/a | Involved in the study | |
|---|---|---|
| ☐ | ☒ | Antibodies |
| ☒ | ☐ | Eukaryotic cell lines |
| ☒ | ☐ | Palaeontology and archaeology |
| ☐ | ☒ | Animals and other organisms |
| ☒ | ☐ | Clinical data |
| ☒ | ☐ | Dual use research of concern |
| ☒ | ☐ | Plants |

### Methods

| n/a | Involved in the study | |
|---|---|---|
| ☒ | ☐ | ChIP-seq |
| ☒ | ☐ | Flow cytometry |
| ☒ | ☐ | MRI-based neuroimaging |

## Antibodies

| | |
|---|---|
| Antibodies used | anti-Fgf7 antibody (Abcam CAT: ab131162)amount/dilution : IF: 1:100 western blotting:1:1000; anti-Acta2 antibody (BioLegend CAT: 904601)IF:1:100 Western blotting: 1:1000; anti-Col1a1 antibody (Abcam CAT: ab34710) amount/dilution : IF:1:200 Western blotting: 1:1000; anti-Gapdh antibody (ABclonal CAT: A19056) amount/dilution : Western blotting:1:1000; anti-Sca1 antibody (Abcam CAT: ab51317)amount/dilution: IF:5 µg/mL; anti-Egr1 antibody (Proteintech CAT: 22008-1-AP) IF:1:100; anti-Ki67 antibody (Abcam CAT: ab16667),IF:1:250; anti-Mkx antibody (lifespan CAT: LSB8063-50),IF:1:250; anti-Lox antibody (HUABIO CAT: RT1367)amount/dilution: IF:1:50; anti-Uts2r antibody (HUABIO CAT: ER1909-98)amount/dilution: IF:1:50; anti-Thbs4 antibody (abcam CAT: ab176116)amount/dilution: IF:1:100; anti-Tnmd (abcam CAT: ab203676)amount/dilution: IF:1:100; anti-Col3 (proteintech CAT: 22734-1-AP)amount/dilution: IF:1:100. |
| Validation | All of the antibodies were validated by the suppliers and our research results.FGF7: https://www.abcam.com/en-us/products/primary-antibodies/kgf-fgf-7-antibody-epr7261-ab131162;ACTA2: https://www.biolegend.com/de-de/products/anti-alpha-smooth-muscle-actin-antibody-11003?GroupID=GROUP756;COL1A1: https://www.abcam.cn/products/primary-antibodies/collagen-i-collagen-iii-antibody-ab34710;GAPDH: https://abclonal.com.cn/catalog/A19056;SCA1: https://www.abcam.cn/products/primary-antibodies/ly-6a-e-sca-1-antibody-e13-161-7-hematopoietic-stem-cell-marker-ab51317;EGR1: https://www.ptgcn.com/products/EGR1-Antibody-22008-1-AP.htm;Ki67: https://www.abcam.cn/products/primary-antibodies/ki67-antibody-sp6-ab16667;MKX: https://www.labome.com/product/LifeSpan-Biosciences/LS-B8063.html ;LOX: https://huabio.cn/products/LOX-1-antibody-RT1367;UTS2R: https://huabio.cn/products/GPR14-antibody-ER1909-98;THBS4: https://www.abcam.cn/products/unavailable/thbs4-antibody-n-terminal-ab176116;TNMD: https://www.abcam.cn/products/primary-antibodies/tenomodulin-antibody-ab203676;COL3: https://www.ptgcn.com/Products/COL3A1-Antibody-22734-1-AP.htm |

## Animals and other research organisms

Policy information about studies involving animals; ARRIVE guidelines recommended for reporting animal research, and Sex and Gender in Research

| | |
|---|---|
| Laboratory animals | The animal experiments were conducted using mice with a C57BL/6J genetic background. Sample collection time point is 7 days after birth, 14 days after birth,21 days after birth,8-10 weeks old; Sprague-Dawley rats, 8 weeks old. |
| Wild animals | This study did not include wild animals. |
| Reporting on sex | To avoid potential impacts on the experimental results due to male aggressive behavior , we used only female rats in the animal models for this study. This study did not consider sex in study design. Due to the occurrence of tendon fibrosis across different physiological sexes, mice were not distinguished by sex in the study. |
| Field-collected samples | This study did not involve samples collected from the field. |
| Ethics oversight | Zhejiang University Institutional Animal Care and Use Committee (ZJU20240291). |

Note that full information on the approval of the study protocol must also be provided in the manuscript.

## Plants

| | |
|---|---|
| Seed stocks | *Report on the source of all seed stocks or other plant material used. If applicable, state the seed stock centre and catalogue number. If plant specimens were collected from the field, describe the collection location, date and sampling procedures.* |
| Novel plant genotypes | *Describe the methods by which all novel plant genotypes were produced. This includes those generated by transgenic approaches, gene editing, chemical/radiation-based mutagenesis and hybridization. For transgenic lines, describe the transformation method, the number of independent lines analyzed and the generation upon which experiments were performed. For gene-edited lines, describe the editor used, the endogenous sequence targeted for editing, the targeting guide RNA sequence (if applicable) and how the editor was applied.* |
| Authentication | *Describe any authentication procedures for each seed stock used or novel genotype generated. Describe any experiments used to assess the effect of a mutation and, where applicable, how potential secondary effects (e.g. second site T-DNA insertions, mosiacism, off-target gene editing) were examined.* |
