# Supplementary material for: FGF7 promotes load-bearing tendon regeneration and suppresses fibrosis
Source: Nat Commun. 2025 Dec 27;17:708. doi: 10.1038/s41467-025-67355-7 (PMC12820144; doi:10.1038/s41467-025-67355-7)
Supplement: Supplementary file 3 — Supplementary Materials [file 41467_2025_67355_MOESM3_ESM.pdf]

Supplementary Materials for

FGF7 promotes load-bearing tendon regeneration and suppresses fibrosis

Ruifu Lin *et al.*

*Corresponding author. Zi Yin, yinzi@zju.edu.cn

This PDF file includes:

Figs. S1 to S20

Tables S1 to S2

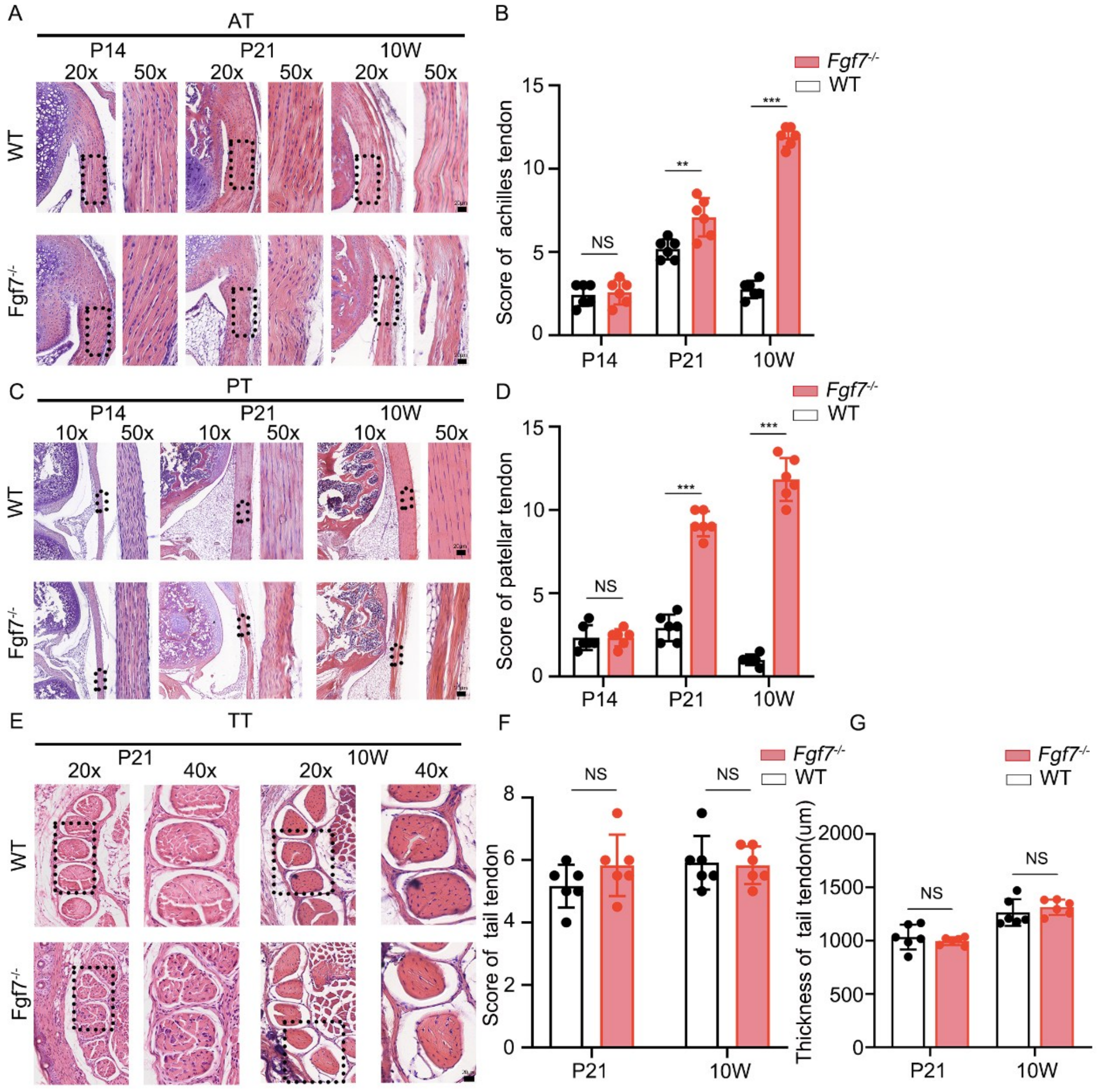

Fig. S1. Histological analysis of load-bearing tendon structures in *Fgf7*$^{-/-}$ and wildtype mice. (A) Hematoxylin and eosin (HE) staining of Achilles tendon at postnatal days 14, 21, and 10 weeks in *Fgf7*$^{-/-}$ and wild-type mice. Scale bars, 20 μm. **(B)** Microscopic-level histological scoring of the Achilles tendon at postnatal days 14, 21, and 10 weeks in *Fgf7*$^{-/-}$ and wild-type mice. (n=6 per group, bars represent mean ± SD; $^{***}P$<0.001, NS: not significant, two-tailed Student's t test). **(C)** Hematoxylin and eosin (HE) staining of patellar tendon at postnatal days 14, 21, and 10 weeks in *Fgf7*$^{-/-}$ and wild-type mice. Scale bars, 20 μm. **(D)** Microscopic-level histological scoring of the patellar tendon at postnatal days 14, 21, and 10 weeks in *Fgf7*$^{-/-}$ and wild-type mice. (n=6 per group, bars represent mean ± SD; $^{**}P$<0.01, $^{***}P$<0.001, NS: not significant, two-tailed Student's t test). **(E)** Hematoxylin and eosin (HE) staining of tail tendon at

postnatal days 14, 21, and 10 weeks in *Fgf7*$^{-/-}$ and wild-type mice. Scale bars, 20 μm. **(F)** Microscopic-level histological scoring of tail tendon at postnatal days 14, 21, and 10 weeks in *Fgf7*$^{-/-}$ and wild-type mice. (*n*=6 per group, bars represent mean ± SD; NS: not significant, two-tailed Student's t test). **(G)** Statistical analysis of tail tendon thickness in HE-stained sections at postnatal days 21, and 10 weeks in *Fgf7*$^{-/-}$ and wildtype mice (*n*=6 per time point, Error bars represent SDs; WT: wildtype. NS: not significant, two-tailed Student's *t* test). Source data are provided as a Source Data file.

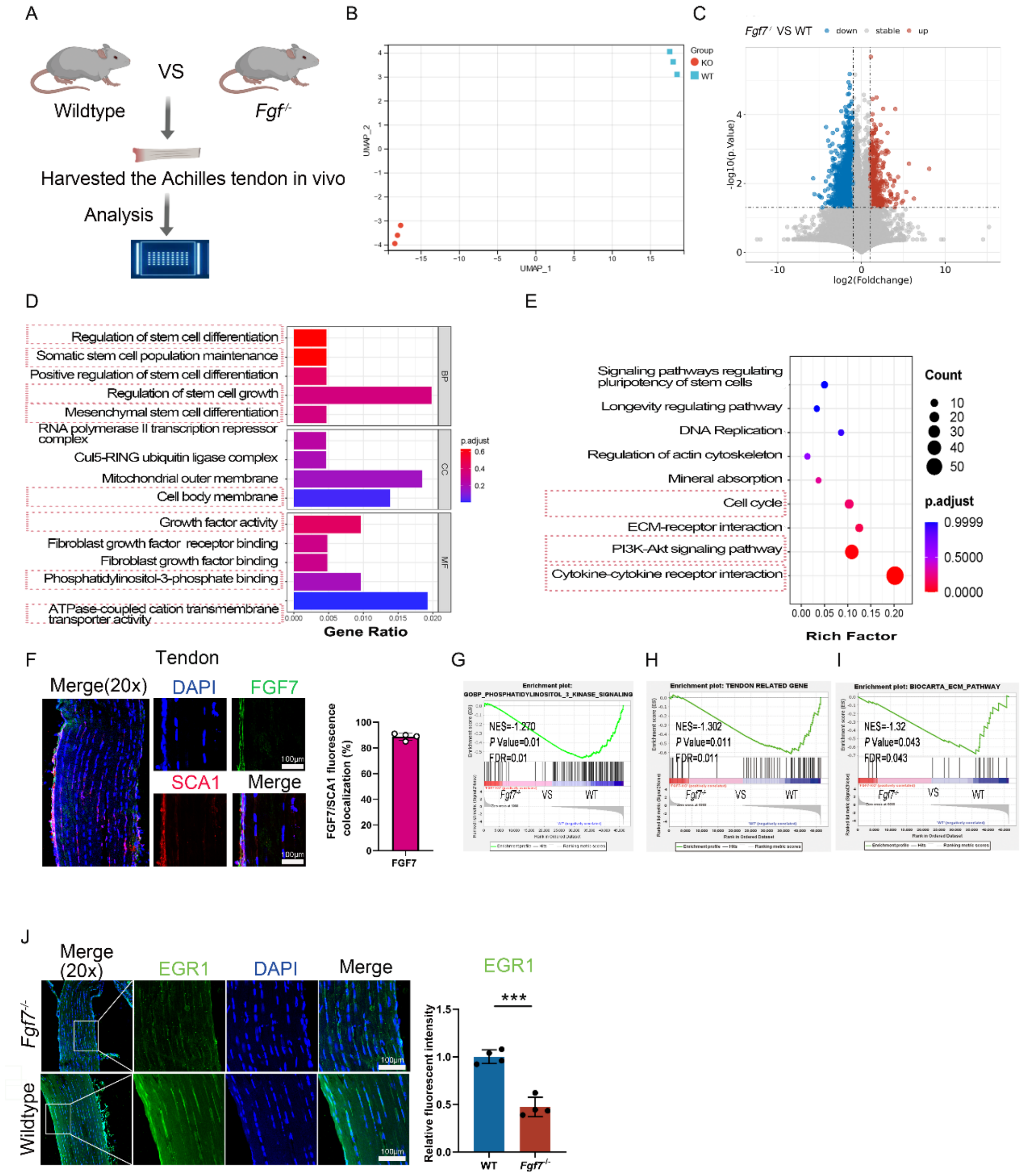

Fig. S2. Altered signaling pathways related to tenocyte function in tendon tissues of *Fgf7*$^{-/-}$ mice. (A) Schematic representation of RNA-Seq sequencing from tendon tissues of *Fgf7*$^{-/-}$ and wildtype mice. **(B)** Principal component analysis (PCA) plot showing clustering of sequencing sample (KO representing *Fgf7*$^{-/-}$ mouse tendons and WT representing wildtype mouse tendons). **(C)** Volcano plot displaying upregulated and downregulated genes in tendon tissues from *Fgf7*$^{-/-}$ versus wildtype mice (up: 287, down: 2210; log2 fold change >1.5, <1.5; p<0.05; n=3 per group). **(D)** KEGG analysis

showing differential expression of multiple stem cell-related pathways in tendon tissues from *Fgf7*$^{-/-}$ and wildtype mice. **(E)** KEGG enrichment analysis displaying key differential signaling pathways between tendon tissues from *Fgf7*$^{-/-}$ and wildtype mice. **(F)** Co-localization immunofluorescence staining of FGF7 protein and SCA1 protein in tendon and epitenon tissues, with corresponding co-localization fluorescence ratio statistics (n=4 independent experiments, bars represent mean ± SD). Scale bars, 100 μm. **(G)** GSEA plot showing differential expression of the PI3K-AKT signaling pathway in tendon tissues from *Fgf7*$^{-/-}$ and wildtype mice. NES: normalized enrichment score. **(H)** GSEA plot showing the differential expression of tendon-related gene sets in tendon tissues of *Fgf7*$^{-/-}$ mice compared to wildtype mice. NES, normalized enrichment score. **(I)** GSEA plot showing the differential expression of the ECM pathway in tendon tissues of *Fgf7*$^{-/-}$ mice compared to wildtype mice. NES, normalized enrichment score. **(J)** Immunofluorescence staining and statistical analysis of the expression levels of the tendon-related marker protein EGR1 in tendon tissues of wildtype and *Fgf7*$^{-/-}$ mice (n=4 independent experiments, bars represent mean ± SD; WT: wildtype. $^{***}P$<0.001, two-tailed Student's t test). Scale bars, 100 μm. Source data are provided as a Source Data file.

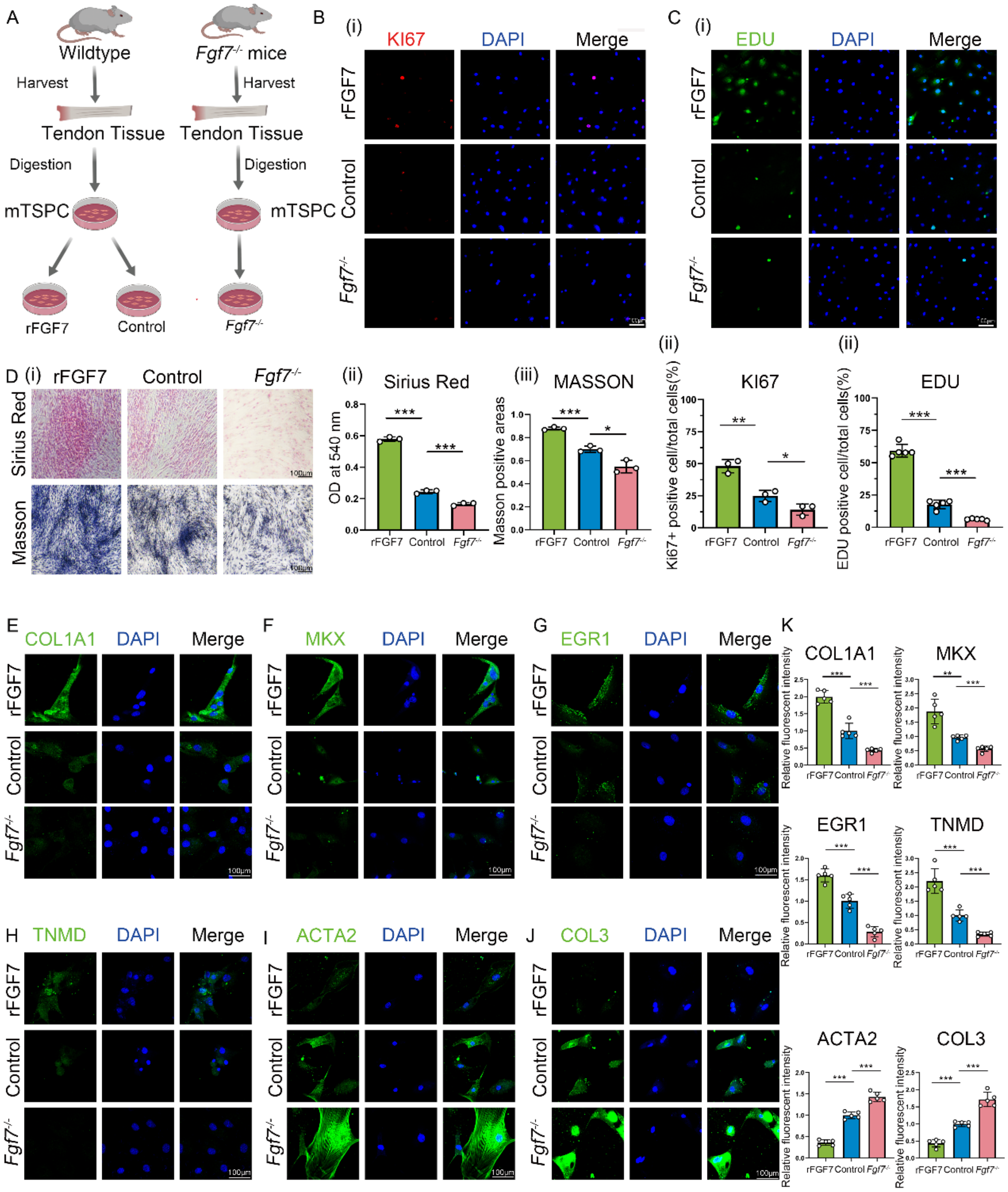

Fig. S3. FGF7 promotes mTSPCs tenogenic differentiation and inhibits fibrotic differentiation. **(A)** Schematic representation of the experimental procedure for promoting tendon lineage differentiation of mTSPCs using FGF7 growth factor(rFGF7). **(B)** Immunofluorescence staining of KI67 protein in mTSPCs treated with rFGF7, PBS, and mTSPCs from wildtype mice or *Fgf7*$^{-/-}$ mice(i) and semi-quantitative fluorescence analysis (ii) (n=3 independent experiments, bars represent mean ± SD; $^{*}P$<0.05, $^{**}P$<0.01, two-tailed Student's t test). Scale bars, 100 μm. **(C)** Immunofluorescence

staining of EDU protein in mTSPCs treated with rFGF7, PBS, and mTSPCs from *Fgf7*$^{-/-}$ mice (i) and semi-quantitative fluorescence analysis(ii) (n=5 independent experiments, bars represent mean ± SD; $^{***}P$<0.001, two-tailed Student's t test). Scale bars, 100 μm. **(D)** Sirius Red and Masson's Trichrome staining of mTSPCs treated with rFGF7, PBS and mTSPCs from *Fgf7*$^{-/-}$ mice after 14 days of tendon lineage culture(i), with Sirius Red optical density (OD) statistical analysis(ii) (n=3, independent experiments, bars represent mean ± SD; $^{***}P$<0.001, two-tailed Student's t test), and statistical analysis of the rate of Masson's Trichrome positive area(iii)(n=3, independent experiments, bars represent mean ± SD; $^{*}P$<0.05, $^{***}P$<0.001, two-tailed Student's t test). Scale bars, 100 μm. **(E-K)** Immunofluorescence staining of tendon-related proteins (COL1A1, MKX, EGR1, TNMD) and fibrotic proteins (ACTA2, COL3) in mTSPCs treated with rFGF7, PBS and mTSPCs from *Fgf7*$^{-/-}$ mice, with corresponding semi-quantitative fluorescence analysis (n=5 independent experiments, bars represent mean ± SD; $^{**}P$<0.01, $^{***}P$< 0.001, two-tailed Student's t test). Scale bars, 100 μm. Source data are provided as a Source Data file.

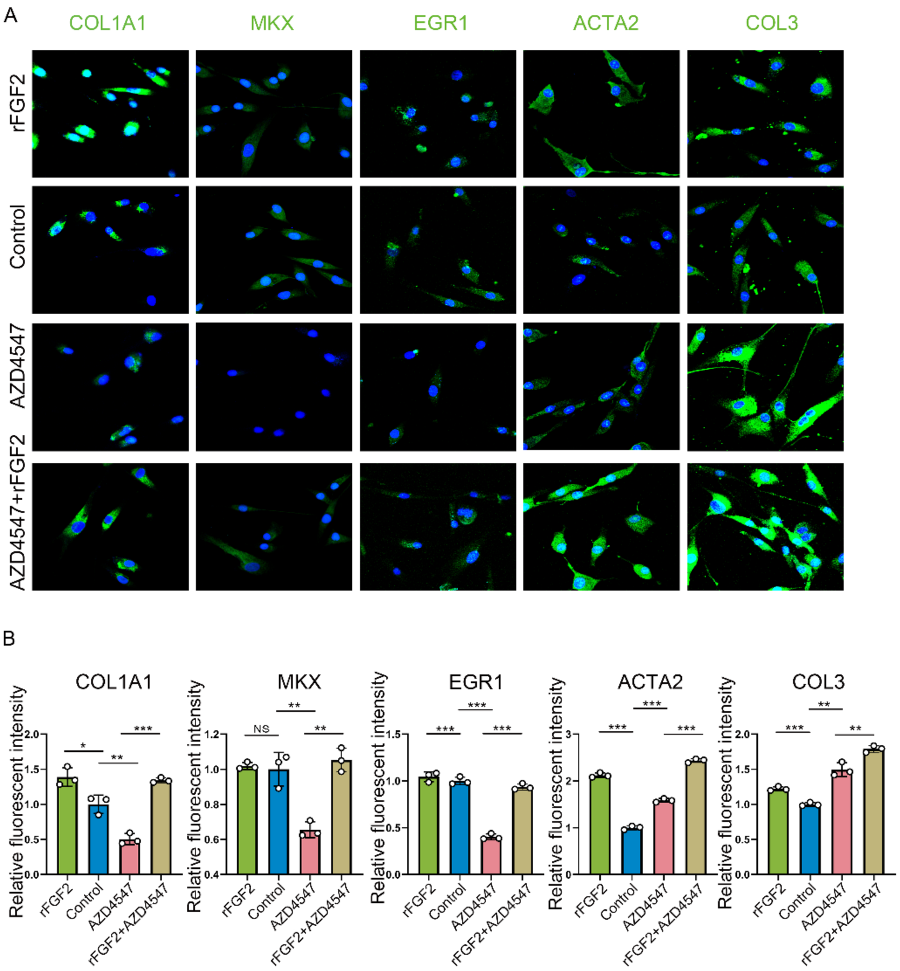

Fig. S4. Effects of recombinant FGF2 on tendon-related markers in hTSPCs. (A-B) Immunofluorescence staining of tendon-related proteins (COL1A1, MKX, EGR1) and fibrotic proteins (ACTA2, COL3) in hTSPCs treated with rFGF2, PBS, AZD4547, co-treated with rFGF2 and AZD4547, corresponding semi-quantitative fluorescence analysis*,* n=3 independent experiments, $^{**}P$<0.01, $^{***}P$<0.001, two-tailed Student's t test, Scale bars, 100 μm. Source data are provided as a Source Data file.

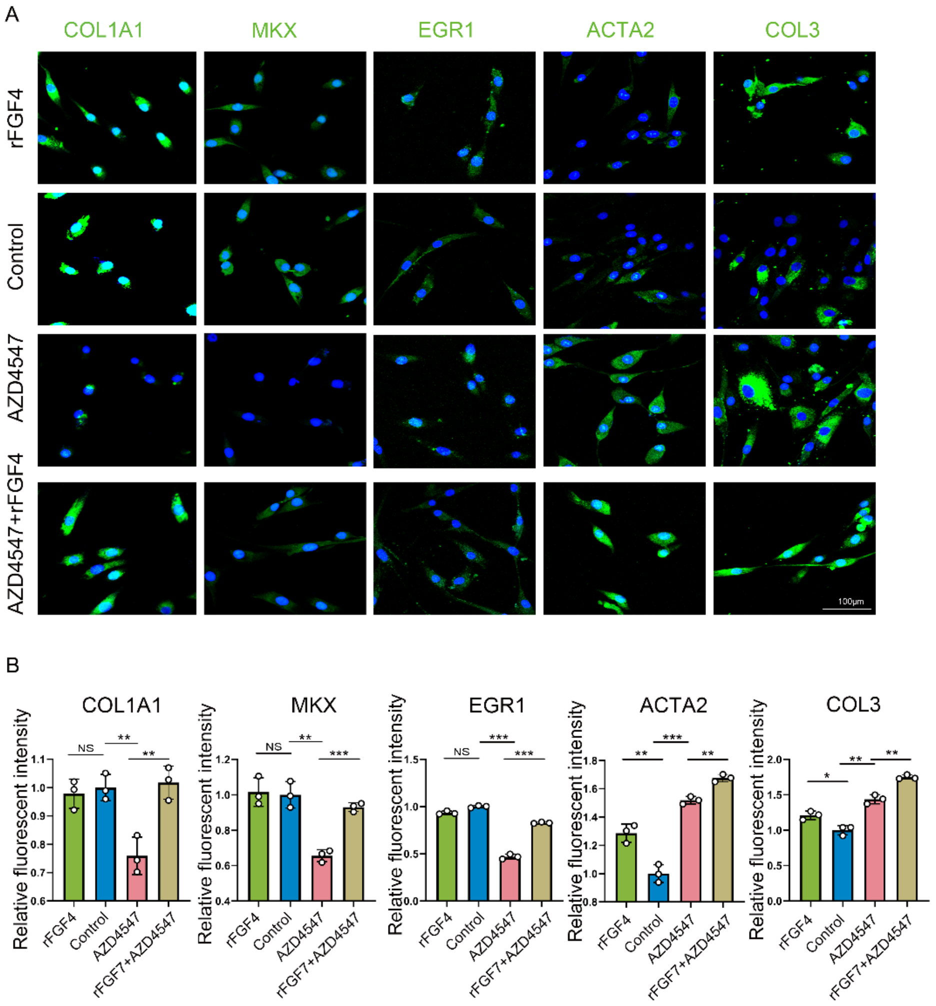

Fig. S5. Effects of recombinant FGF4 on tendon-related markers in hTSPCs. (A-B) Immunofluorescence staining of tendon-related proteins (COL1A1, MKX, EGR1) and fibrotic proteins (ACTA2, COL3) in hTSPCs treated with rFGF4, PBS, AZD4547, co-treated with rFGF4 and AZD4547, corresponding semi-quantitative fluorescence analysis*, n*=3 independent experiments, $^{**}P$<0.01, $^{***}P$<0.001, two-tailed Student's *t* test, Scale bars, 100 μm. Source data are provided as a Source Data file.

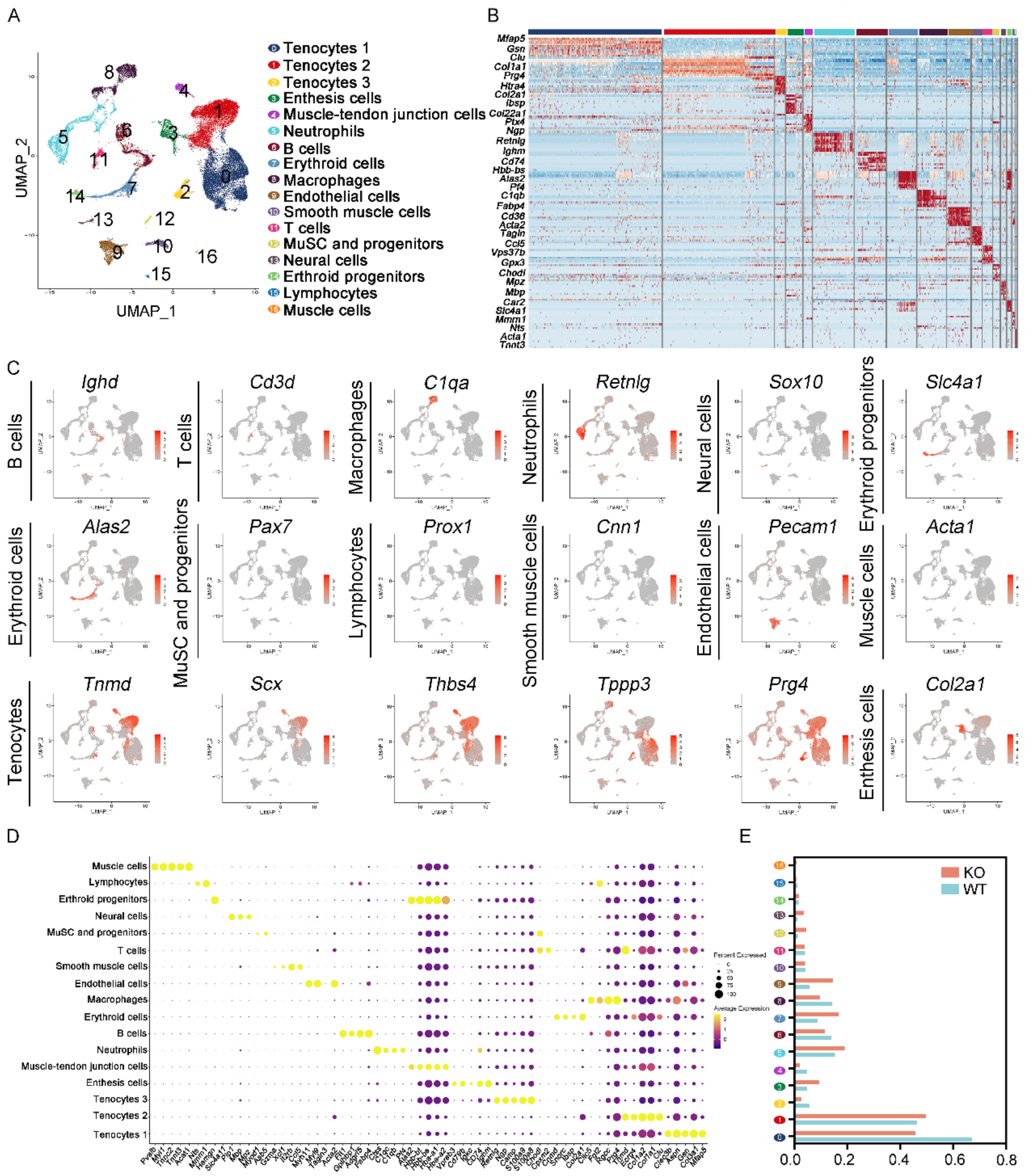

Fig. S6. Overview of single-cell transcriptome profiling on tendons from *Fgf7*$^{-/-}$ and wildtype mice. (A) Uniform Manifold Approximation and Projection (UMAP) plot displaying 31,756 cells divided into 17 distinct subpopulations. **(B)** Heatmap showing the expression of the top 10 marker genes for each of the 17 subpopulations. The gene names are listed on the left, with two representative marker genes highlighted for each subpopulation. The color bar denotes the scaled gene expression value. **(C)** UMAP plots indicating the expression of specific marker genes for various cell subpopulations: B cells (*Ighd*), T cells (*Cd3d*), macrophages (*C1qa*), neutrophils (*Retnlg*), neural cells (*Sox10*), erythroid progenitors (*Slc4a1*), erythroid cells (*Alas2*),

MuSC and progenitors (*Pax7*), lymphocytes (*Prox1*), smooth muscle cells (*Cnn1*), endothelial cells (*Pecam1*), muscle cells (*Acta1*), tenocytes (*Tnmd, Scx, Thbs4, Tppp3, Prg4*) and enthesis cells (*Col2a1*). **(D)** Dot plot illustrating the representative differentially expressed genes (DEGs) for each subpopulation. The size of each dot correlates with the percentage of cells expressing the specific gene, while the color intensity corresponds to the average expression level of the gene. **(E)** Representative bar plots showing the percentage of cells belonging to each of the 17 subpopulations in the *Fgf7*$^{-/-}$ and wildtype groups.

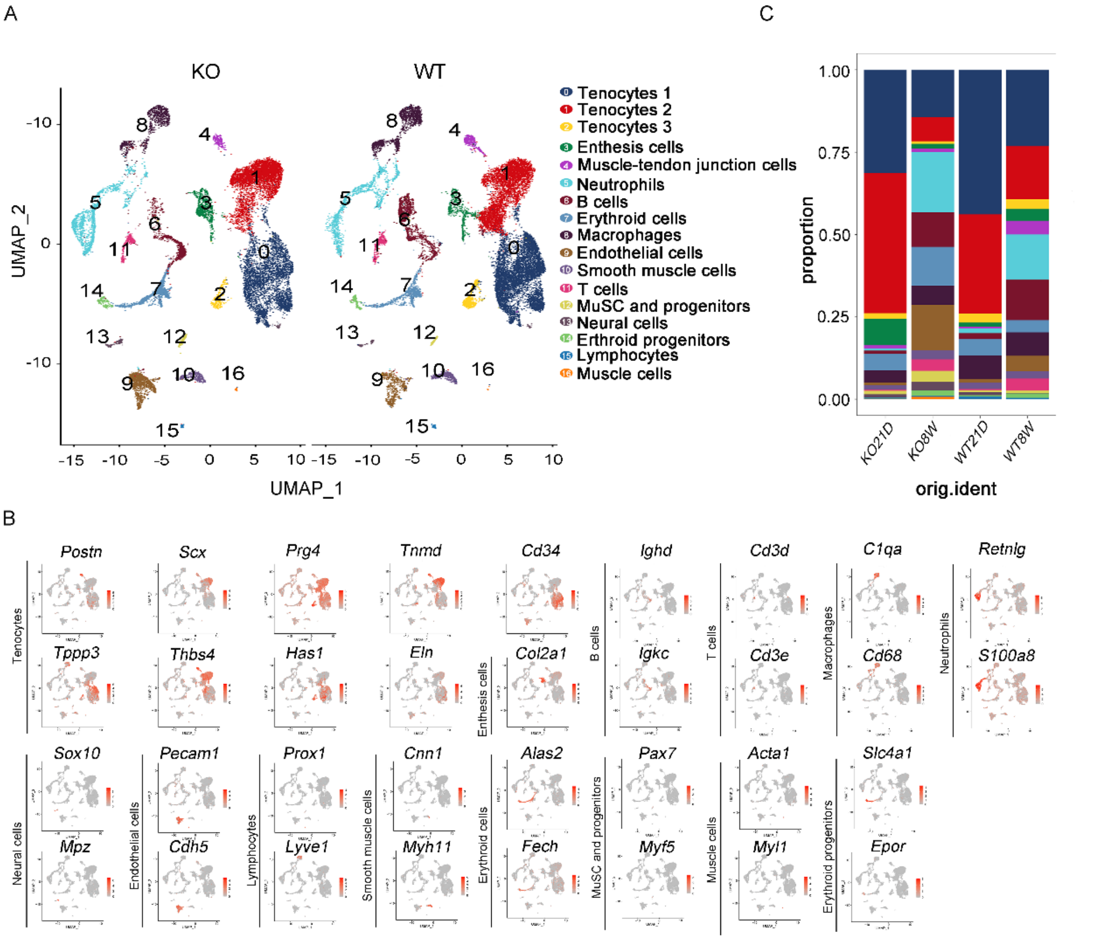

Fig. S7. Overview of single-cell transcriptome profiling of tendons from *Fgf7*$^{-/-}$ and wildtype mice. (A) Uniform Manifold Approximation and Projection (UMAP) plot displaying 22,374 cells from wildtype mouse tendons and 19,055 cells from *Fgf7*$^{-/-}$ mouse tendons, grouped into 17 distinct subpopulations and the representative bar plots showing the percentage of cells belonging to each subpopulation in tendon tissues from four different sources: *Fgf7*$^{-/-}$ mice at 21 days(21d), *Fgf7*$^{-/-}$ mice at 8 weeks (8w), wildtype mice at 21 days(21d) and wildtype mice at 8 weeks(8w). **(B)** UMAP plots indicating the expression of specific marker genes for various cell subpopulations: tenocyte subpopulations(*Postn*, *Scx*, *Prg4*, *Tnmd*, *Cd34*, *Tppp3*, *Thbs4*, *Has1*, *Eln*), enthesis cells (*Col2a1*), B cells (*Ighd*, *Ighc*), T cell (*Cd3d*, *Cd3e*), macrophages (*C1qa*, *Cd68*), neutrophils (*Retnlg*, *S100a8*), erythroid progenitor cell subpopulation (*Slc4a1*, *Epor*), neural cells (*Sox10*, *Mpz*), endothelial cells (*Pecam1*, *Cdh5*), lymphocytes (*Prox1*, *Lyve1*), smooth muscle cells (*Cnn1*, *Myh11*), erythroid cells (*Alas2*, *Fech*), MuSC and progenitors (*Pax*, *Myf5*) and muscle cells (*Acta1*, *Myl1*). **(C)** Representative

bar plots showing the percentage of cells belonging to each subpopulation in tendon tissues from four different sources: *Fgf7*$^{-/-}$ mice at 21 days(21d), *Fgf7*$^{-/-}$ mice at 8 weeks (8w), wildtype mice at 21 days(21d) and wildtype mice at 8 weeks(8w).

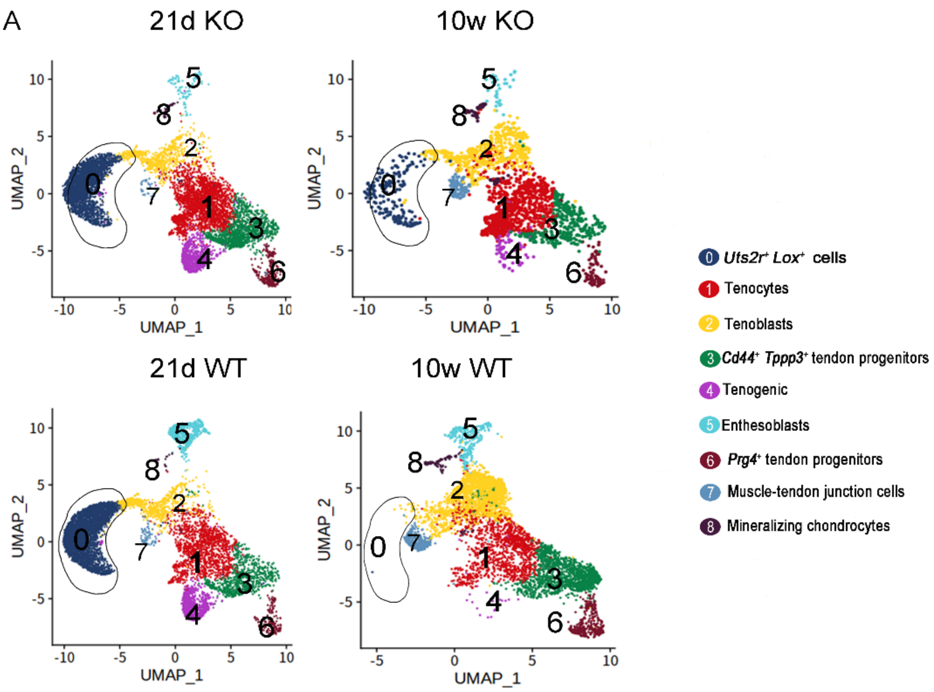

Fig. S8. ***Uts2r*****[+]*****Lox*****[+] cell persistence in** ***Fgf7*****[-/-] adult tendon. (A)** UMAP plots showing the distribution of the 9 subclusters of Achilles tendon samples from 21 days *Fgf7*[-/-] mice, 8-week *Fgf7*[-/-] mice, 21 days wildtype mice, 8-week wildtype mice.

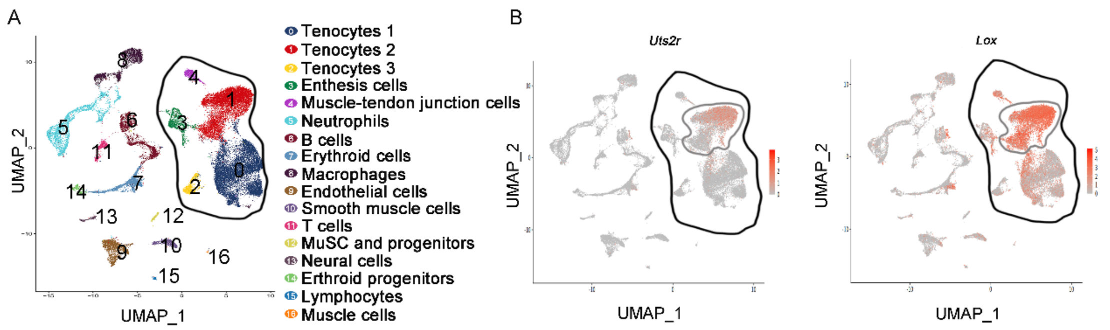

Fig. S9. Localization of a *Lox*⁺*Uts2r*⁺ subcluster within tendon cell subpopulation. (A) Uniform Manifold Approximation and Projection (UMAP) plot displaying 31,756 cells divided into 17 distinct subpopulations. The circle subpopulation includes subgroups 0, 1, 2, 3, and 4, which exhibit tendon characteristics. **(B)** UMAP plots indicating the expression of *Lox*$^{+}$*Uts2r*$^{+}$ subcluster is primarily concentrated within the tendon characteristics subpopulation. The grey circle highlights Tenocytes 2, where the *Lox*$^{+}$*Uts2r*$^{+}$ subcluster is predominantly expressed.

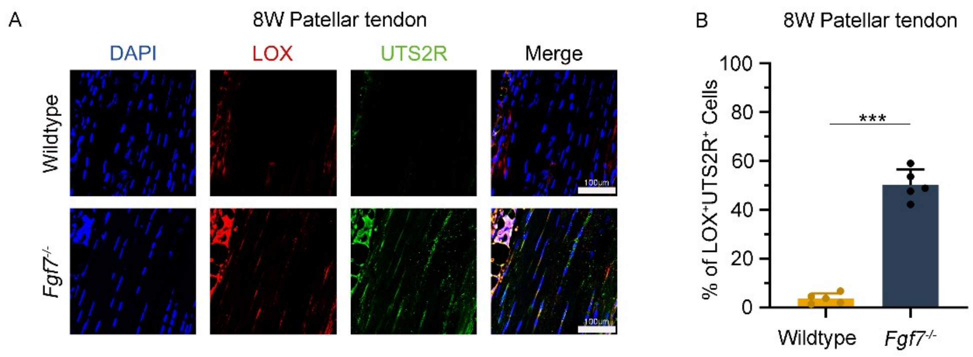

Fig. S10. *Lox*$^{+}$*Uts2r*$^{+}$ subcluster in patellar tendon tissues. (A) Confocal fluorescence imaging showing *Lox*$^{+}$*Uts2r*$^{+}$ subcluster in patellar tendon tissues from 8-week *Fgf7*$^{-/-}$ and wildtype mice. **(B)** The statistical analysis of *Lox* and *Uts2r* co-expression with corresponding statistical analysis (n=5 independent experiments, bars represent mean ± SD; $^{***}P$<0.001, two-tailed Student's t test). Scale bars,100μm. Source data are provided as a Source Data file.

A

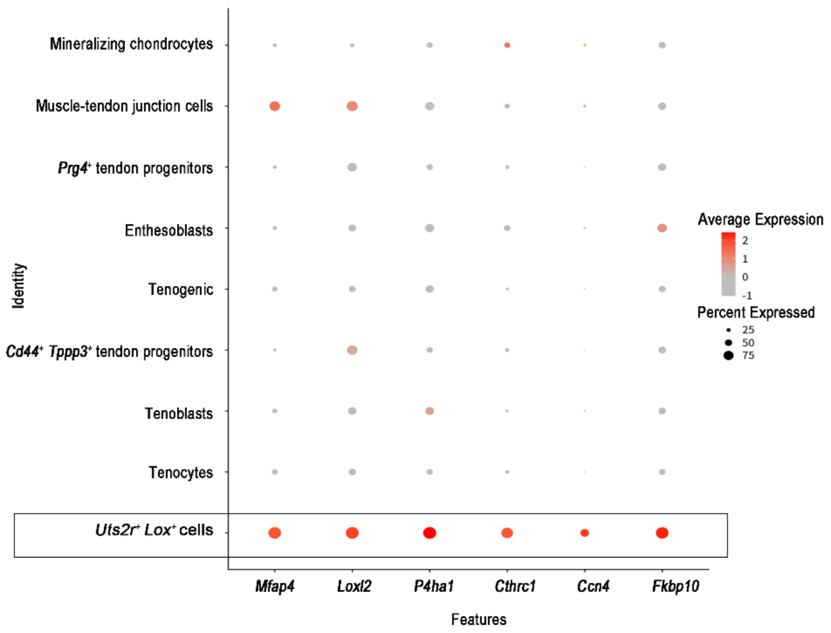

Fig. S11. The dot plot of the fibrosis-related genes in 9 subclusters from 8-week *Fgf7*$^{-/-}$ and 8-week wildtype mice Achilles tendon samples. (A) Dot plot showing selected promoting collagen fiber formation and cross-linking genes (*Mfap4*, *Loxl2*, *P4ha1*, *Cthrc1*, *Ccn4*, *Fkbp10*) enriched in the *Lox*$^{+}$*Uts2r*$^{+}$ subpopulation.

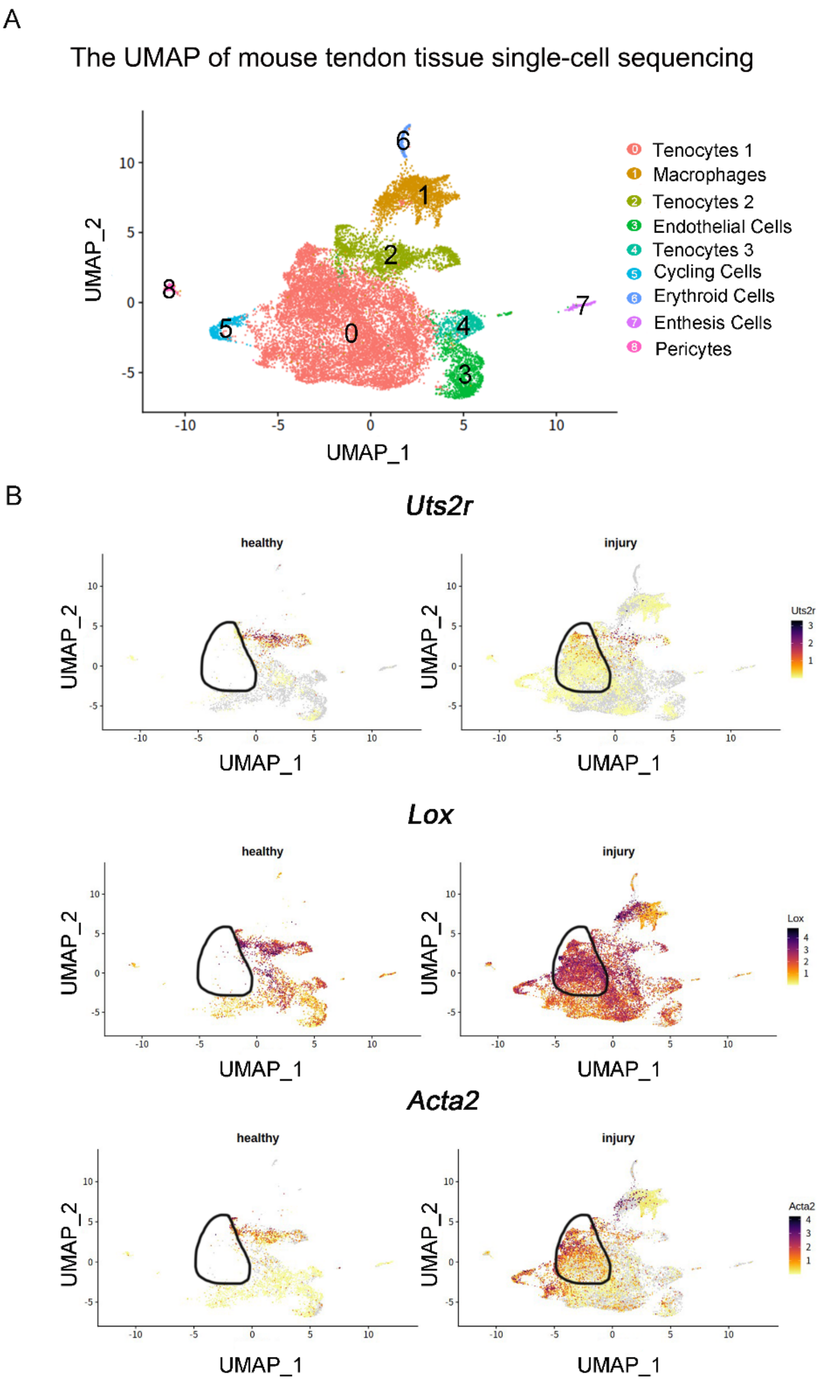

Fig. S12. Profibrotic *Lox*$^{+}$*Uts2r*$^{+}$ cell population emerges and expands post-injury in tendon. (A). A Uniform Manifold Approximation and Projection (UMAP) plot is presented divided into 9 distinct subpopulations. **(B).** UMAP plots illustrating the expression of matrix assembly disorder marker genes *Lox*, *Uts2r*, *Acta2*. The subpopulation within the black circle represents the increased number of cells following injury, where the *Lox*$^{+}$*Uts2r*$^{+}$ cell subpopulation shows elevated expression.

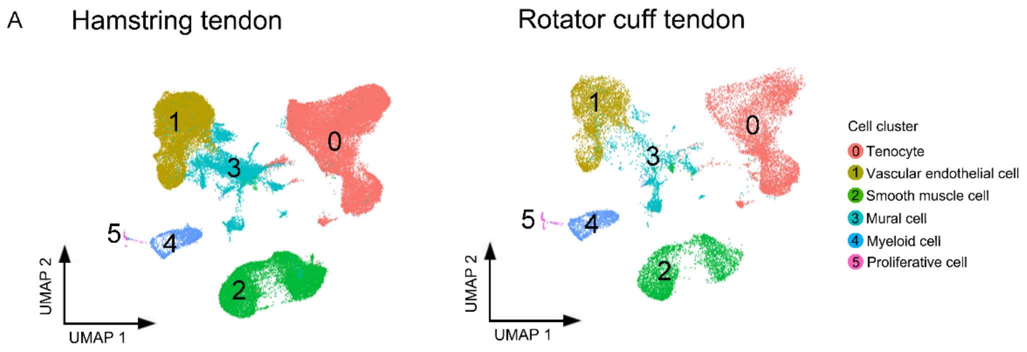

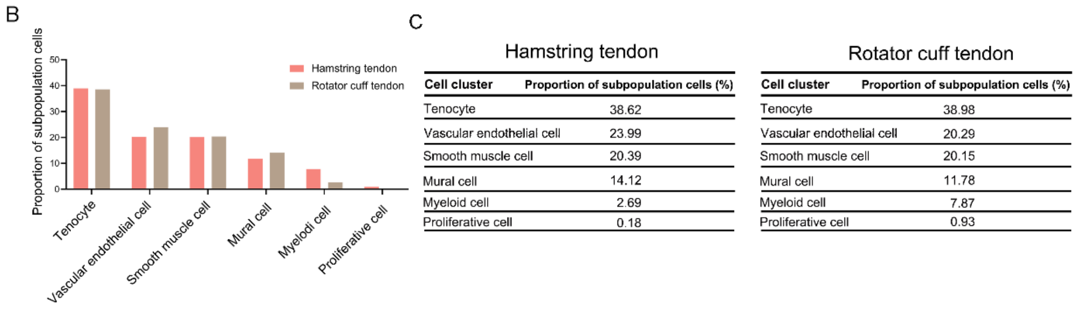

C

Hamstring tendon

Cell cluster	Proportion of subpopulation cells (%)
Tenocyte	38.62
Vascular endothelial cell	23.99
Smooth muscle cell	20.39
Mural cell	14.12
Myeloid cell	2.69
Proliferative cell	0.18

Rotator cuff tendon

Cell cluster	Proportion of subpopulation cells (%)
Tenocyte	38.98
Vascular endothelial cell	20.29
Smooth muscle cell	20.15
Mural cell	11.78
Myeloid cell	7.87
Proliferative cell	0.93

Fig. S13. Cellular composition of human rotator cuff and hamstring tendons. (A). UMAP plots of single-cell sequencing data from normal rotator cuff and normal hamstring tendon tissues. **(B-C).** Statistical data on the proportions of each cell subpopulation, along with specific numerical values for each proportion.

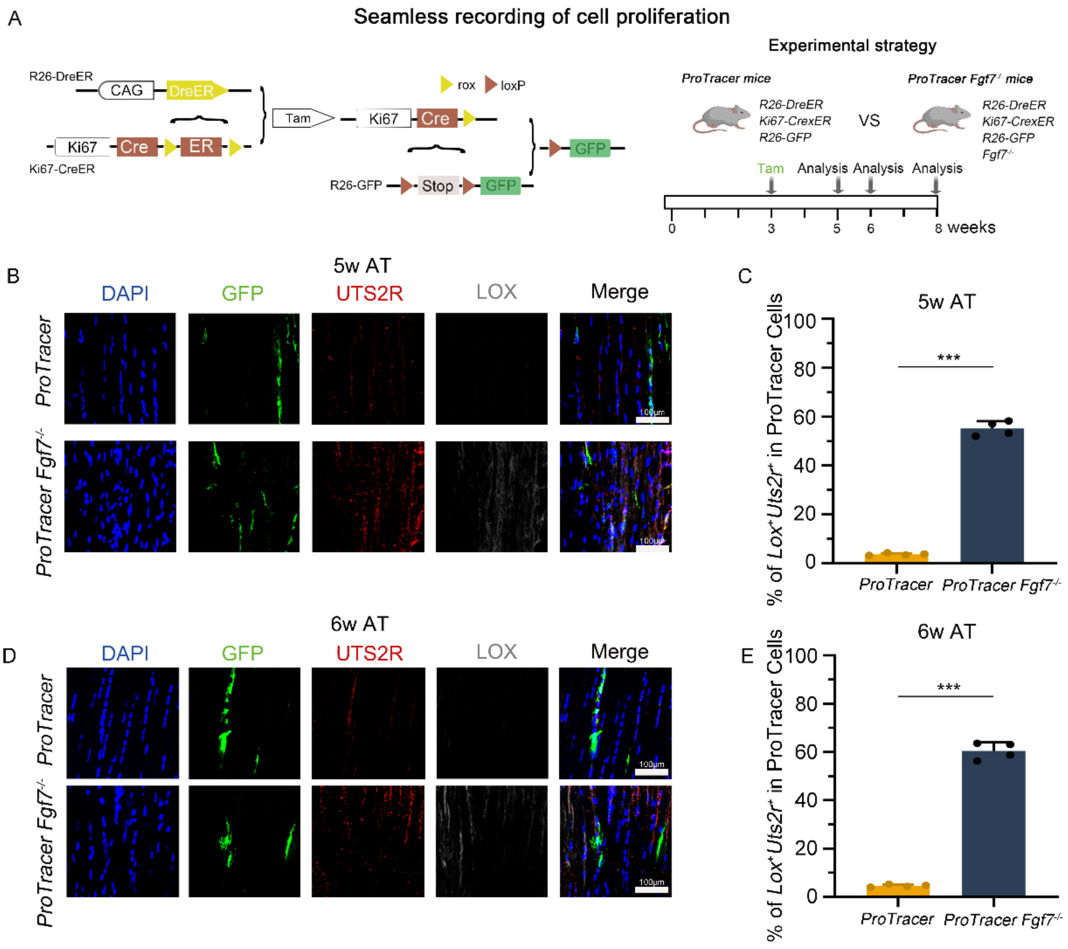

Fig. S14. The observation of *GFP*+*Lox*+*Uts2r*+ cells at multi-time-point. (A) Strategy for the seamless recording of cell proliferation and the schematic of the experimental design. **(B-C)** Confocal fluorescence staining showing co-expression of GFP, UTS2R and LOX in tendon tissues from ProTracer mice and *Fgf7*$^{-/-}$ ProTracer mice at 5 weeks, with corresponding statistical analysis of the proportion of ProTracer labeled cells differentiating into *Lox*$^+$*Uts2r*$^+$ cells (n=4 independent experiments, bars represent mean ± SD; $^{***}P$<0.001, two-tailed Student's t test). Scale bars, 100 µm. **(D-E)** Confocal fluorescence staining showing co-expression of GFP, UTS2R and LOX in tendon tissues from ProTracer mice and *Fgf7*$^{-/-}$ ProTracer mice at 6 weeks, with corresponding statistical analysis of the proportion of ProTracer labeled cells differentiating into *Lox*$^+$*Uts2r*$^+$ cells (n=4 independent experiments, bars represent mean ± SD; $^{***}P$<0.001, two-tailed Student's t test). Scale bars, 100 µm. Source data are provided as a Source Data file.

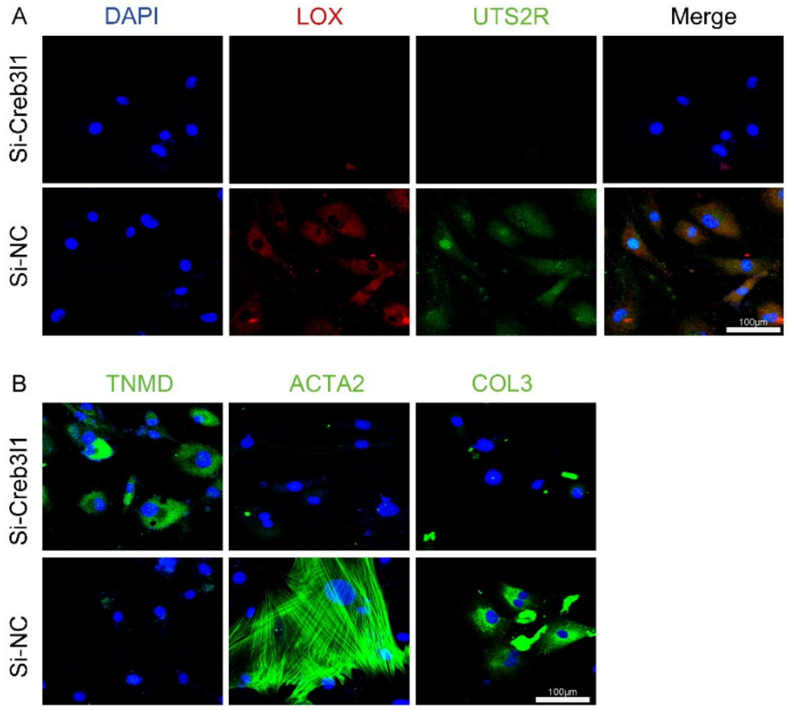

Fig. S15. Effects of *Creb3l1* knockdown in *Fgf7*$^{-/-}$ mTSPCs. (A) Immunofluorescence staining of LOX and UTS2R in mTSPCs from *Fgf7*$^{-/-}$ mice treated with Si-*Creb3l1* or Si-*NC*, Scale bars, 100 μm. **B.** Immunofluorescence staining of tendon-related proteins (TNMD) and fibrotic proteins (ACTA2, COL3) in mTSPCs from *Fgf7*$^{-/-}$ mice treated with Si-*Creb3l1* or Si-*NC*, 100 μm.

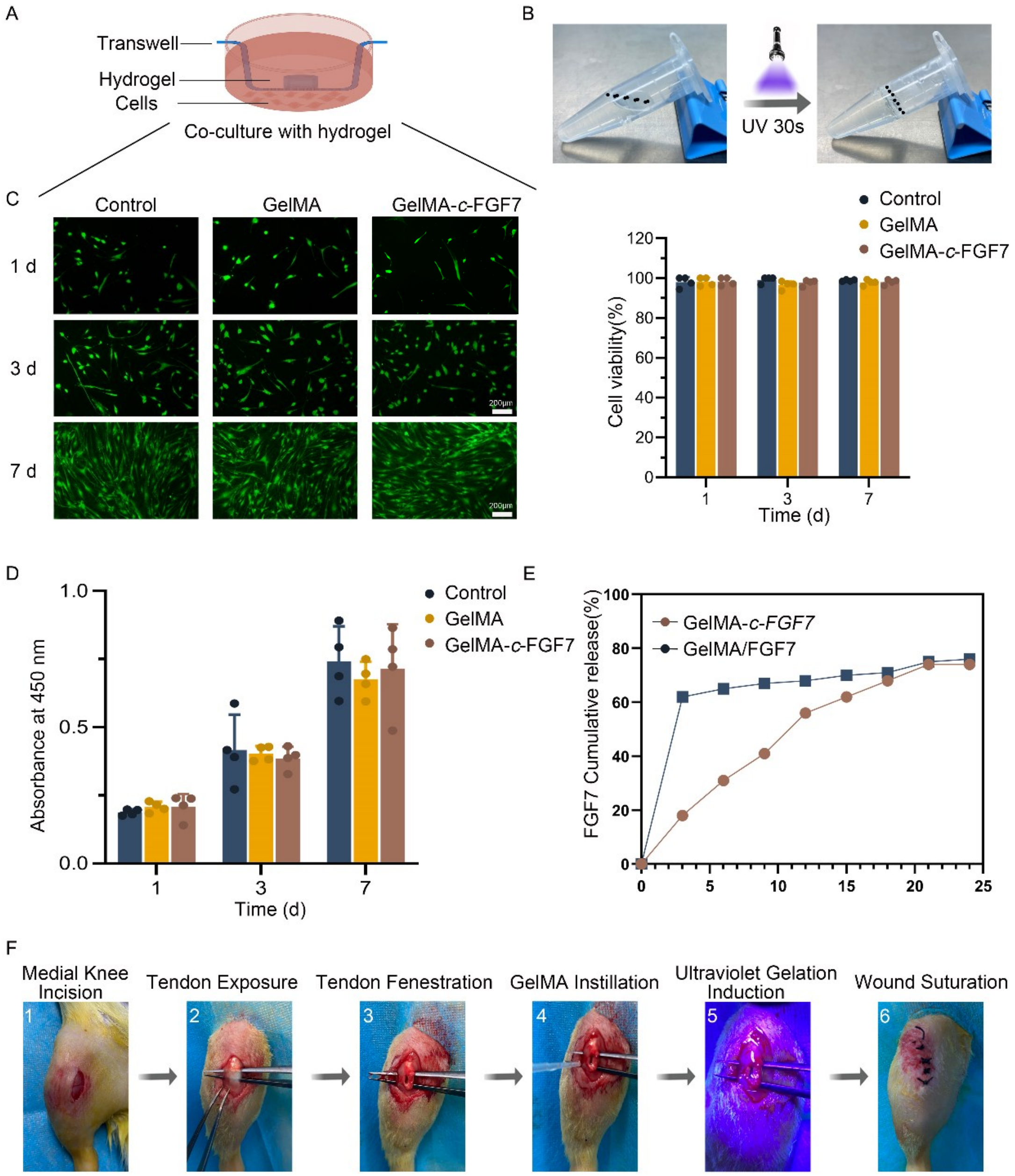

Fig. S16. Biocompatibility and bioactivity characterization of GelMA-*c*-rFGF7 and patellar tendon fenestration modeling with GelMA-*c*-rFGF7 application. **(A)** Schematic representation of hydrogel co-culture and live/dead cell analysis. **(B)** Formation of GelMA-*c*-rFGF7 solution and hydrogel after UV light exposure. **(C)** Study of cell viability over 7 days of co-culture with hydrogel and rat cells. (n=4, independent experiments, bars represent mean ± SD). Scale bars, 200 μm. **(D)** Cell Counting Kit-8 (CCK-8) assay for rat tenocytes co-cultured with hydrogel over 7 days.

(*n*=4, independent experiments, bars represent mean ± SD). **(E)** Cumulative protein release from GelMA-*c*-rFGF7 and GelMA/FGF7. **(F)** Schematic of the patellar tendon fenestration modeling and application of GelMA-*c*-rFGF7 for repair: Step 1: Lateral knee incision; Step 2: Tendon exposure; Step 3: Scaffold preparation; Step 4: Tendon fenestration; Step 5: GelMA instillation; Step 6: Ultraviolet gelation induction; Step 7: Suturation. Source data are provided as a Source Data file.

A

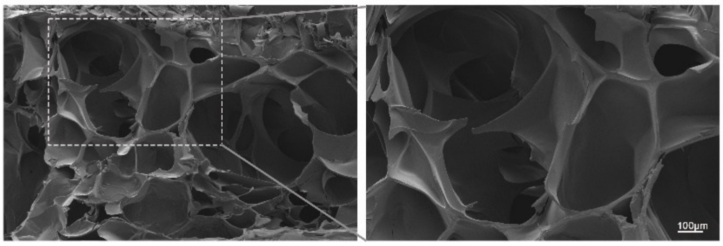

Fig. S17. Scanning electron microscope (SEM) images for hydrogel. A. Representative scanning electron microscope (SEM) images, scale bars: 100μm.

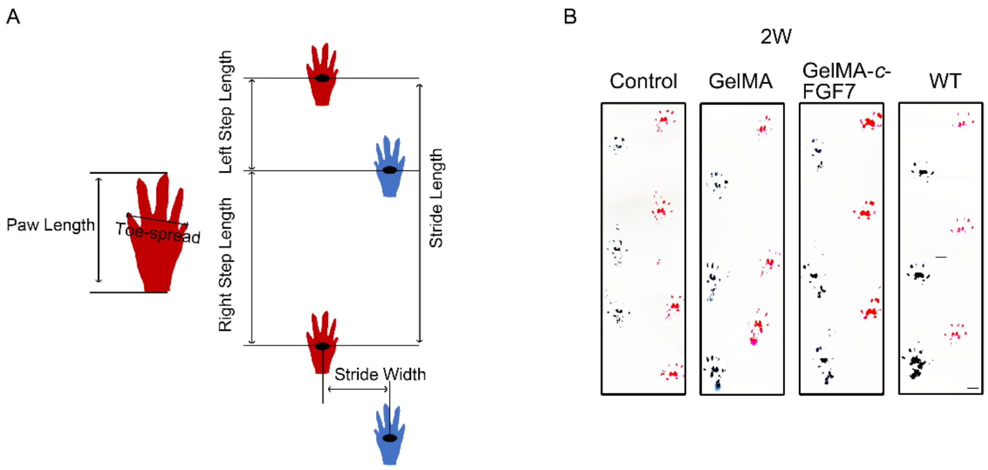

Fig. S18. Motor behavior analysis on rats. (A) Schematic diagram of paw and gait parameters. **(B)** Representative paw prints of each group at 2 weeks post-surgery. Scale bars, 5 mm.

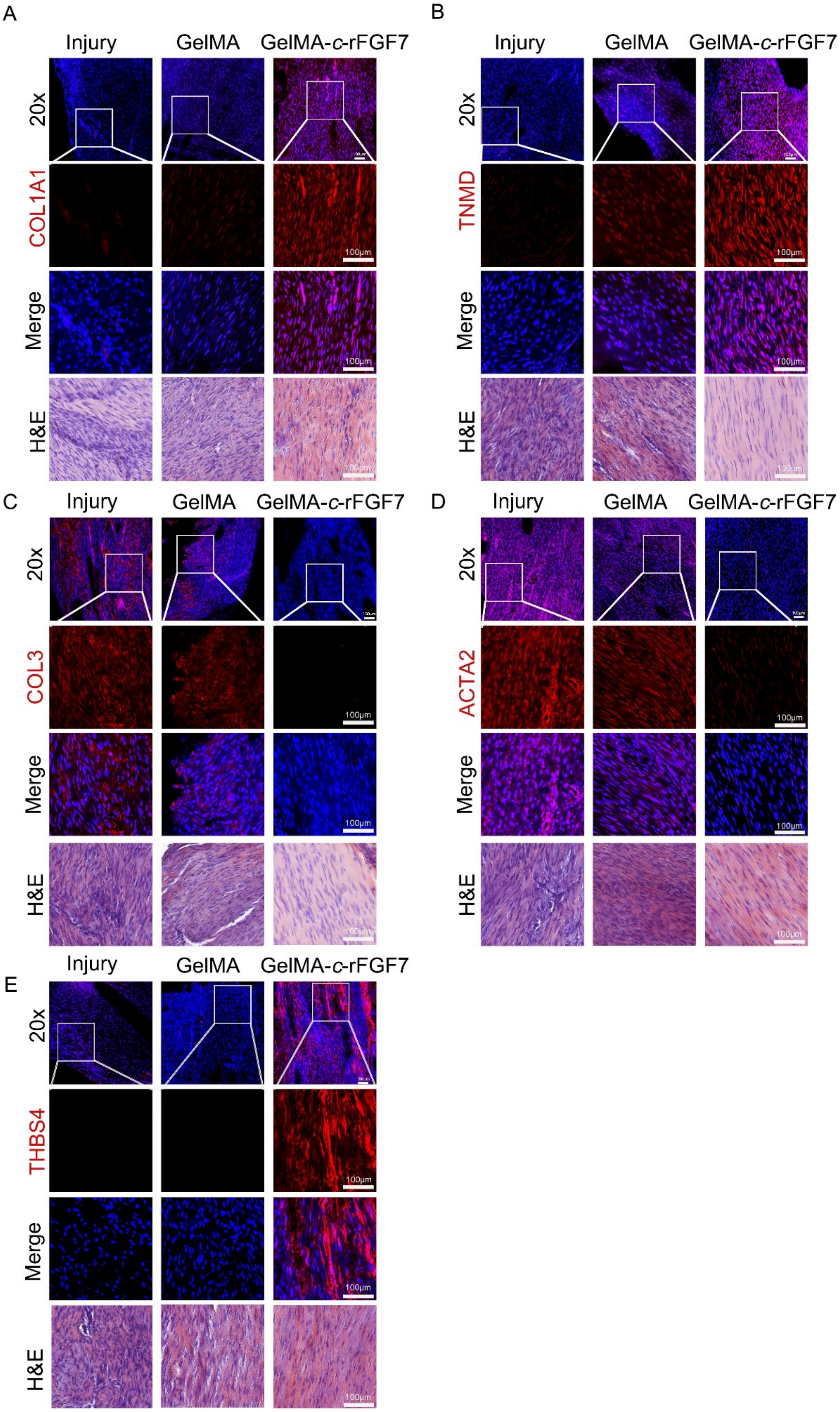

Fig. S19. The observation of representative H&E staining and

immunofluorescence staining at the repair site at 4 weeks post-surgery for each group (Injury, GelMA, GelMA-*c*-rFGF7). (A-E) Representative H&E staining and immunofluorescence staining for tendon-related proteins (COL1A1, TNMD, THBS4) and fibrosis-related proteins (COL3, ACTA2) at the repair site at 4 weeks post-surgery for each group (Injury, GelMA, GelMA-*c*-rFGF7). Scale bars, 100 μm.

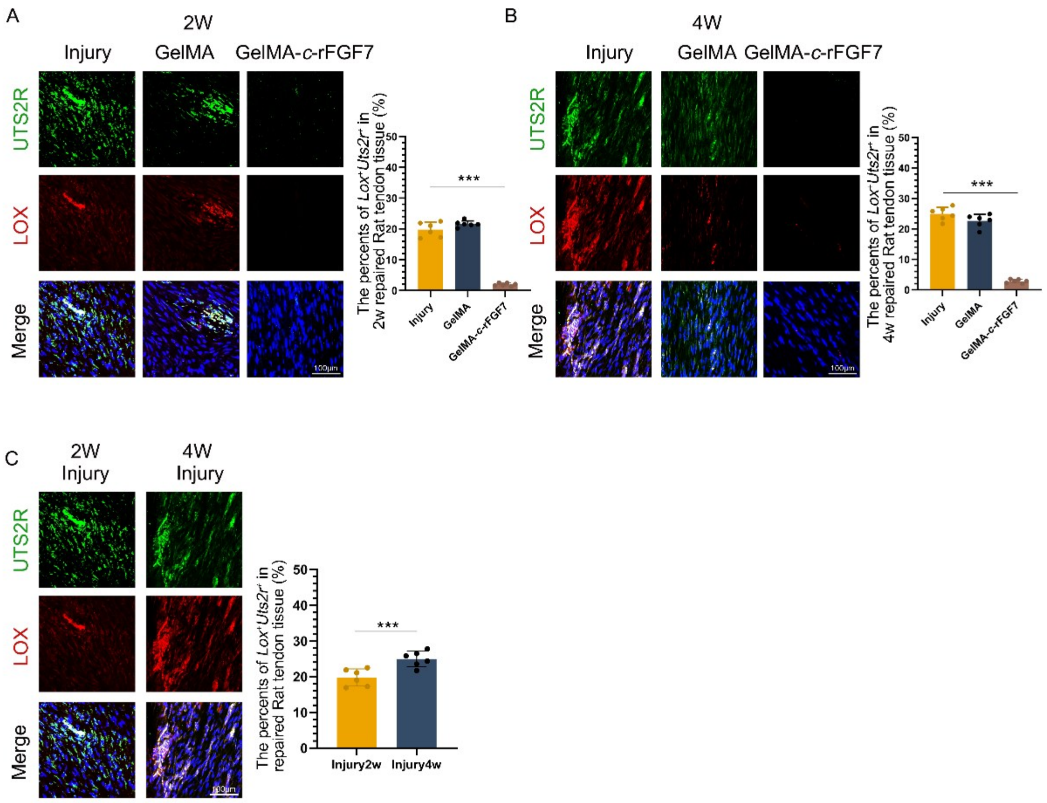

Fig. S20. Expression of *Uts2r*⁺ *Lox*⁺ cells during the repair process of injured rat patellar tendons treated with GelMA-*c*-rFGF7 hydrogel, GelMA, and the untreated injury group. (A) Confocal fluorescence imaging showing the co-expression of *Lox* and *Uts2r* at 2 weeks post-surgery for each group (Injury, GelMA, GelMA-*c*-rFGF7), *n*=6 independent experiments, bars represent mean ± SD; $^{***}P$<0.001, two-tailed Student's *t* test. Scale bars, 100 μm. **(B)** Confocal fluorescence imaging showing the co-expression of *Lox* and *Uts2r* at 4 weeks post-surgery for each group (Injury, GelMA, GelMA-*c*-rFGF7), *n*=6 independent experiments, bars represent mean ± SD; $^{***}P$<0.001, two-tailed Student's *t* test. Scale bars, 100 μm. **(C)** $Uts2r^{+}Lox^{+}$ cells gradually increase during the tendon injury process in rats from 2 weeks to 4 weeks post-injury. *n*=6 independent experiments, bars represent mean ± SD; $^{***}P$<0.001, two-tailed Student's *t* test. Scale bars, 100 μm. Source data are provided as a Source Data file.

Table S1. The primer sequences used for RT-qPCR assays.

Genes	5'-3'	Primer
Human *COL1A1*	Forward	GTGCTAAAGGTGCCAATGGT
	Reverse	ACCAGGTTCACCGCTGTTAC
Human *MKX*	Forward	TCAAGGACAACCTCGGCCTG
	Reverse	ACGGGTTGTCACGGTGCTTG
Human *BGN*	Forward	GGACTCTGTCACACCCACCT
	Reverse	CTTGTTGTTCACCAGGACGA
Human *GAPDH*	Forward	CCATCTTCCAGGAGCGAGATC
	Reverse	GCCTTCTCCATGGTGGTGAA
Mouse *Mkx*	Forward	GACTCCGAGGCTCTGCCGCAA
	Reverse	CAGGAGTCGCCATCGCTGCTCA
Mouse *Egr1*	Forward	ATGGCAGCGGCCAAGGCC
	Reverse	GGGTACGGTTCTCCAGACCCT
Mouse *Dcn*	Forward	CACCCGACACAACCTTGCTAG
	Reverse	GCCTTTCCAACTTCACGAGAGG
Mouse *Tnc*	Forward	CCTGTCCCAATGACTGCAGC
	Reverse	GGTACTCAGTGACCCGCATC
Mouse *Col1a1*	Forward	TGACTGGAAGAGCGGAGAGT
	Reverse	GTTCGGGCTGATGTACCAGT
Mouse *Col3*	Forward	GCCTCCCAGAACATTACATAC
	Reverse	CAATGTCATAGGGTGCGATA
Mouse *Acta2*	Forward	ACTACTGCCGAGCGTGAGAT
	Reverse	CTTCTCCAGGGAGGAAGAGG
Mouse *Thbs4*	Forward	AACCTGAGACCATTGAATTGAGG
	Reverse	CTGCCGGTTAAAGTCCCCTG
Mouse *Tnmd*	Forward	TGGGTGGTCCCTCAAGTGAAAGT
	Reverse	CTCGACGGCAGTAAATACAACAATA
Mouse	Forward	CCATCTTCCAGGAGCGAGATC

Gapdh	Reverse	GCCTTCTCCATGGTGGTGAA
Mouse	Forward	TGGCCTCCAGCTACATTTCT
Scx	Reverse	TGTCACGGTCTTTGCTCAAC

Table S2. Antibodies used for IF staining and Western blotting.

Antibodies	Company	Code	Applications
FGF7	abcam	ab131162	IF, Western blotting
ACTA2	BioLegend	904601	IF, Western blotting
COL1A1	abcam	ab34710	IF, Western blotting
GAPDH	ABclonal	A19056	Western blotting
SCA1	abcam	ab51317	IF
EGR1	Proteintech	22008-1-AP	IF
KI67	abcam	ab16667	IF
MKX	lifespan	LSB8063-50	IF
LOX	HUABIO	*RT1367*	IF
UTS2R	HUABIO	*ER190998*	IF
THBS4	abcam	ab176116	IF
TNMD	abcam	ab203676	IF

COL3	proteintech	22734-1-AP	IF
